# Molecular mechanisms underlying the control of antigenic variation in African trypanosomes

**DOI:** 10.1016/j.mib.2010.08.009

**Published:** 2010-12

**Authors:** David Horn, Richard McCulloch

**Affiliations:** 1 London School of Hygiene and Tropical Medicine, Keppel Street, London, WC1E 7HT, UK; 2 University of Glasgow, Institute of Infection, Immunity and Inflammation and Wellcome Trust Centre for Molecular Parasitology, Glasgow Biomedical Research Centre, 120 University Place, Glasgow, G12 8TA, UK

## Abstract

African trypanosomes escape the host adaptive immune response by switching their dense protective coat of Variant Surface Glycoprotein (VSG). Each cell expresses only one *VSG* gene at a time from a telomeric expression site (ES). The ‘pre-genomic’ era saw the identification of the range of pathways involving *VSG* recombination in the context of mono-telomeric *VSG* transcription. A prominent feature of the early post-genomic era is the description of the molecular machineries involved in these processes. We describe the factors and sequences recently linked to mutually exclusive transcription and *VSG* recombination, and how these act in the control of the key virulence mechanism of antigenic variation.

## Introduction

**Figure 1:**
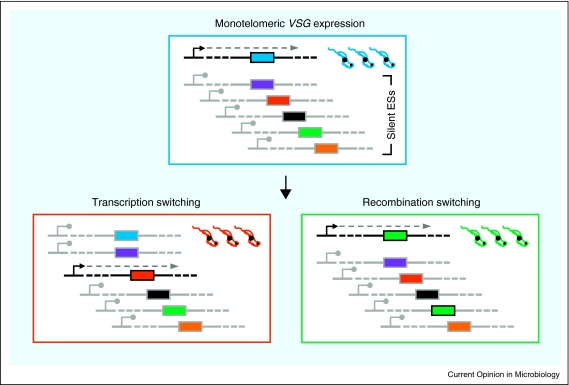
The schematic illustrates mono-telomeric *VSG* expression and routes of *VSG* switching. NB: there are >1000 *VSG* (pseudo)genes available for the exchange or assembly of new *VSGs* at the active ES; most of these are in long subtelomeric arrays flanked by repetitive elements.

Many pathogens have evolved strategies for phenotypic and clonal variation of surface proteins. This allows for the establishment of a persistent infection in immunocompetent hosts, enhancing transmission. The African trypanosome, *Trypanosoma brucei*, is one such pathogen; a protozoan of major medical and economic importance. These highly motile cells circulate in the mammalian host bloodstream and are spread by tsetse flies. Evasion of the adaptive host immune response is achieved by changing the composition of a dense Variant Surface Glycoprotein (VSG) coat on bloodstream form cells [1]. The VSG is invariably encoded in a polycistronically transcribed telomeric expression site (ES). Importantly, *VSG* expression is monoallelic such that only one among 10–20 telomeric ESs is transcribed at a time [2]. Silencing at all other ESs maintains monoallelic expression and the integrity of the evasion strategy while the multiplicity of potential ESs (Figure 1) allows for a co-ordinated switch to transfer active transcription from one telomere to another [3]. Beyond the *VSG*s found in the ES, *T. brucei* also possesses a massive archive of ∼1000 silent *VSG*s and *VSG* pseudogenes which dominate subtelomeres [27]. Recombination is therefore central to antigenic variation, allowing the parasite to utilise this *VSG* archive, typically by copying a different gene into the active ES (Figure 1).

Recent reviews have dealt with a range of topics related to antigenic variation in *T. brucei*, focusing on DNA breaks as triggers for recombination-based switching [4], expression-site associated genes [5], VSG expression patterns and mechanisms [6] and the trafficking and barrier function of the VSG coat [7,8]. Here, we focus on recent advances in understanding the molecular machineries that maintain *VSG* allelic exclusion and that execute recombination-based *VSG* switching.

## Control of monoallelic *VSG* expression site transcription

The epigenetic mechanisms underlying *VSG* gene silencing and allelic exclusion are of great intrinsic scientific interest and also present potential targets for chemotherapy. Subtelomeric promoters and genes are typically prone to silencing in a range of organisms, a phenomenon first described in yeast [9] and subsequently demonstrated in trypanosomes [10,11^•^]. Crucially, in *T. brucei*, only one of the available bloodstream ESs [2] must specifically escape silencing to maintain homogeneity of the VSG coat and the ability to rapidly swap exposed epitopes; the resulting differential in *VSG* expression between silent and active ESs may be in excess of 10,000-fold. A notable feature of *VSG* ESs is transcription by RNA polymerase I (RNAPI). Although all *VSG* ES promoters appear to initiate RNAPI-mediated transcription at a similar rate, transcription is processive only at the single ‘active’ *VSG* ES [12] and this ES associates with an extranucleolar accumulation of RNAPI known as the ES body (ESB) [13]. It remains unknown whether the ESB self-assembles around the active gene [14], or whether the structure excludes other ESs [13]. Thus, the mechanism allowing one ES to escape silencing remains something of a mystery, and no ESB-specific factor has been identified to date, but there has been some recent progress in understanding the structure and behaviour of the active ES. Nucleosomes are depleted at the active ES [15^•^,16^•^], thereby reflecting either transcription-based ejection and/or another form of destabilisation. In addition, sister chromatid cohesion promotes inheritance of the active state; in cells depleted for cohesin components, cohesion at the active ES is compromised leading to an increased rate of transcription switching to alternative telomeres [17^•^]. These studies, and the DOT1B work described below, may provide some early insight into the elusive mechanism of cross-talk that operates among the active and silent ESs.

**Figure 2:**
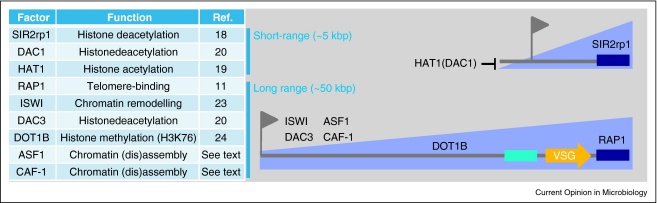
Factors that impact telomeric and *VSG* expression site silencing in *T. brucei*. The role of each factor in the table on the left is illustrated in the schematic on the right. Only the SIR2rp1 and RAP1 effects have been shown to diminish as distance from the telomere increases. The short-range effects have only been shown to affect *de novo* telomeres but may also impact *VSG* ES transcription, particularly at the short monocistronic ESs used in the insect mid-gut and during the establishment of a mammalian infection [3]. It is also important to note that these factors could impact recombination. Many of the factors shown were originally named based on phenotypes identified in yeast: ASF, anti-silencing factor; DOT, disruptor of telomeric silencing; SIR, silent information regulator; RAP, repressor/activator protein. The DAC3 homologue in yeast (Hda1p) has also been linked to telomeric exclusion of genes encoding surface-exposed proteins [50,51]. Flags represent promoters and blue boxes represent repetitive sequences; dark, T_2_AG_3_, light, 70 bp.

The powerful silencing mechanism itself has been more readily amenable to investigation and at least six factors required to maintain ES silencing have been identified in recent years. It has also become clear that other factors participate in a distinct form of silencing defined by the distance that the effect spreads from the telomere (Figure 2). ‘Short-range’ telomeric silencing is restricted to a region immediately adjacent to the telomeric repeats and in *T. brucei*, the distal ES promoters and antigenic variation escape this effect. This silencing mechanism requires SIR2rp1, the only nuclear NAD-dependent histone deacetylase in *T. brucei* [18]. Furthermore, the histone acetyltransferase, HAT1 [19], and a histone deacetylase, DAC1 [20^•^], appear to regulate SIR2rp1 spreading. These latter findings reinforce the parallels with telomeric silencing in yeast where the putative homologues, Sas2 [21] and Rpd3 [22], control the spreading of Sir2-dependent silencing.

Substantial evidence has emerged recently to also link chromatin structure and modification to the more extensive ‘long-range’ *VSG* ES silencing (Figure 2). The chromatin chaperones, CAF-1 and ASF1, are required for inheritance of the silent state, presumably through cycles of nucleosome (dis)assembly associated with DNA replication (Alsford *et al*., unpublished). In addition, several enzymes have been identified that are likely to stabilise the nucleosomes at silent sites thereby repressing transcription. These include a chromatin remodeler, ISWI [23], a histone deacetylase, DAC3 [20^•^] and a histone (H3K76) methyltransferase, DOT1B [24^•^]. Most of the factors above are essential for growth and have been depleted using RNA interference. Only the methyltransferase is dispensable and cells lacking this factor display a relatively subtle *VSG* transcription derepression phenotype [24^•^]; the essential factors reveal a more pronounced derepression phenotype at the promoter that, nonetheless, due to attenuation, does not lead to detectable *VSG* expression from the ‘silent’ ESs. This may reflect a more pronounced role at the promoter or simply stasis associated with only partial alteration of the chromatin through the long polycistronic ES. Thus, current results suggest that chromatin modifiers and remodelers cooperate to reinforce and propagate the silent state. Specifically, the silent sites are likely to comprise hypoacetylated, hypomethylated (H3K76, the DOT1B methyltransferase effect is thought to be indirect) and ordered chromatin. The viable *dot1b* methyltransferase mutants also presented an opportunity to investigate the impact on transcription switching to an alternative telomere and these cells displayed a substantial delay in this process [24^•^].

Evidence indicating a role for the telomere itself in *VSG* ES silencing comes from studies on repressor/activator protein 1 (RAP1). This telomere-associated protein is also essential for growth, and RNA interference-based knockdown produced cells expressing multiple VSGs on the surface [11]. RAP1 may recruit SIR2rp1 and additional factors, thereby mediating short-range telomeric silencing, as in yeast [25] and long-range *VSG* ES silencing (see Figure 2). Intriguingly, sustained *VSG* ES silencing at a chromosome end lacking RAP1-binding sites, the terminal telomeric repeats [26], may indicate the presence of silent compartments containing subtelomere clusters.

Telomeres and chromatin are central to tightly regulating interaction between the nuclear pool of RNA polymerase and *VSG* genes. It seems likely that the silencing mechanism targets all telomeres in the context of a dominant, and currently mysterious, anti-silencing machine or factor that compels *VSG* ESs to obey the rules of allelic exclusion. This latter activity apparently acts in a telomere-specific manner. A better understanding of the exclusion process might reveal targets that can be exploited for therapy. Indeed, at least one of the factors linked to silencing, the DAC3 deacetylase, represents a potentially druggable target [20^•^].

## Control of *VSG* expression site recombination

Subtelomeres tend to be ‘fragile sites’ that are prone to rapid gene turnover and increased rates or sequence exchange. As such, these hotbeds of innovation are ideal sites for contingency genes such as *VSGs* [3]. *VSG* switching by recombination most commonly occurs by gene conversion reactions that copy a silent *VSG* into the active ES, replacing the *VSG* that was previously transcribed (Figure 1). This mitotic process requires considerable mechanistic flexibility, since gene conversion reactions have been documented using donor *VSG*s from three distinct genomic locations: the silent ESs, the telomeres of African trypanosome-specific minichromosomes, and from the subtelomeric *VSG* arrays [3]. Gene conversion of array *VSG*s can recombine complete genes into the ES, or can generate novel *VSG*s (‘mosaics’) by segmental gene conversion reactions using multiple *VSG* pseudogenes [27]. A role for homologous recombination (HR) [28] in these gene conversion processes was first revealed by mutating RAD51, the key enzyme of homology-directed DNA strand exchange, resulting in impaired *VSG* switching [29]. Subsequent analysis confirmed the importance of RAD51-directed strand exchange. Mutation of at least one of four *T. brucei* RAD51 paralogues also impairs switching [30] (R Dobson *et al*., unpublished), as does mutation of the *T. brucei* orthologue of BRCA2 [31^•^], a breast cancer oncogene that co-ordinates loading of RAD51 onto processed DNA double strand breaks (DSBs) [28]. In part, this is mediated through conserved BRC repeats, which are dramatically expanded in *T. brucei* BRCA2 [31^•^]; possibly an example of adaptations imposed on the HR machinery by *VSG* switching. An important recent development is the use of a yeast meganuclease (I-*Sce*I) for the controlled generation of a chromosomal DSB [32^•^]; at a chromosome internal locus this results in a temporal cascade of cell cycle stalling, accumulation of RAD51 in subnuclear foci and predominant allelic HR. The system has also now been used for genetic dissection of chromatin control of DSB repair (DSBR) in *T. brucei* (Glover *et al*., unpublished); a histone acetyltransferase and a histone deacetylase have been shown to impact DNA resection and RAD51 filament disassembly respectively.

Beyond the HR strand exchange step, we have much to learn about the upstream and downstream processes in *VSG* switching, and alternative recombination pathways. 70 bp repeats are a key element of *VSG* switching as they flank >90% of VSGs [27], meaning they can provide upstream sequence homology for recombination as well as ES-specific initiation. Recent work suggested an initiating role for DSBs at 70 bp repeats upstream of the active *VSG* [33^•^]: naturally occurring breaks were detected in this region, and the generation of an I-*Sce*I-induced DSB adjacent to the 70 bp repeats increased the rate of switching. A key factor in the detection of DSBs and in nucleolytic processing to allow RAD51 filament formation is the Mre11-Rad50-Xrs2/Nbs1 (MRX) complex [34]. Mutation of MRE11 in *T. brucei* reveals a role in HR repair [35,36], but not in *VSG* switching [35], meaning *VSG* switch-initiating breaks may not be DSBs, at least initially, or that other factors assume an MRX-like function during *VSG* switching.

A number of pathways may contribute to *VSG* switching (Table 1, reviewed in [6]) but the recombination steps that operate downstream of RAD51-mediated strand exchange have been explored to only a limited extent. Break-induced replication (BIR) has gained prominence recently [37], at least in part because of roles in telomere maintenance in yeast and mammals [38]. BIR, involving telomere-proximal *VSG*s and associated 70 bp repeats, might be an adaptation of backup telomere maintenance pathways to satisfy the demands of *VSG* switching [33^•^,39]. However, a mechanistic demonstration of BIR, rather than gene conversion, in *VSG* switching is still needed [40]. For instance, the DNA polymerases (Pols) that catalyse DNA synthesis during *VSG* switching remain unknown. Though HR in eukaryotes relies on Pols α, δ and ɛ, B family replication enzymes [41], other work has suggested roles for Y family Pols in recombination [42].

RAD51-mediated HR is clearly important in antigenic variation, but RAD51-independent pathways also operate [29,30,31^•^]. Deletions based on microhomology-mediated end-joining (MMEJ, aka micro-single-strand annealing) are readily detectable in *T. brucei* following an I-*Sce*I-induced DSB [32^•^] and one-sided, MMEJ-based gene conversion also operates (Glover *et al*., unpublished). MMEJ has also been observed in *T. brucei* cell extracts [43] and following DNA transformation [44] and occurs in mutants lacking RAD51 and KU, a key component of non-homologous end-joining (NHEJ). The relationship between MMEJ in *T. brucei* and alternative end-joining (A-EJ) pathways in other eukaryotes [45] is currently unclear. Nevertheless, though MMEJ/A-EJ is considered subservient to NHEJ in other eukaryotes, significant roles in immunoglobulin gene rearrangements have been described [46]. In addition, evidence is emerging that MMEJ/A-EJ can be a significant route for chromosome rearrangements [47], a process that can shape genomes and may be suppressed by NHEJ [48]. Indeed, MMEJ may be coupled to BIR in such rearrangements [49]. Though KU70-80 is conserved, the Ligase IV-XRCC4 complex of NHEJ has not been found in the trypanosomatids, so these parasites may have evolved to enhance MMEJ.

## Conclusions

Mono-telomeric *VSG* expression and recombination are central to the process of antigenic variation in African trypanosomes and it will be important to understand the machinery underlying both of these processes. The recent work highlighted above has begun to illuminate both areas with chromatin and epigenetics emerging as prominent features. Additional regulators will emerge and further studies on interactions, mapping of epigenetic marks and their functional analysis, nuclear location and cell-cycle control will provide further insights while forward genetic approaches may reveal further novelty and could shed some light on the machinery required for mutually exclusive escape from silencing. Further characterization of DSB processing, HR and MMEJ, their contribution to the various *VSG* recombination reactions and their genetic dissection, are also needed. Finally, now the importance of the terminal telomeric repeats is established for gene silencing, other subtelomeric sequences may be found to serve cis-regulatory functions in spreading heterochromatin, serving as transcription boundaries or enhancers or promoting instability and recombination.

## References and recommended reading

Papers of particular interest, published within the period of review, have been highlighted as:

• of special interest

## Figures and Tables

**Table 1:** DSBR pathways and their possible contribution to antigenic variation in *T. brucei*.

Pathway	Sub-pathway	Features	Proposed role in *VSG* switching	Ref.
Homologous recombination (HR)	Gene conversion (GC)	Copying and replacement of a segment of DNA using flanking homologies	Any VSG (fragment) with sufficient homology could be copied into the active ES by this typically RAD51-dependent mechanism^a^	[29]

	Single-strand annealing (SSA)	Deletion of a segment of DNA using flanking homologies	None	

	Break-induced replication (BIR)	Copying and replacement of a segment of DNA to the chromosome end using a single region of homology	The subset of telomeric *VSGs* could be copied into the active ES by this typically RAD51-dependent mechanism^a^	[33]

End-joining (EJ)	Non-homologous EJ (NHEJ)	Re-ligation of broken strands typically with small deletions	None — not seen in *T. brucei*	

	Microhomology-mediated EJ (MMEJ, aka micro-SSA)	Deletion of a segment of DNA using flanking microhomologies of 5–20 bp. Gene conversion (see above) can be mediated by one-sided MMEJ	MMEJ-based equivalents of (one-sided) GC and BIR would be predicted to be RAD51-dependentand independent respectively^a^ (see above)	[32]

a Recombination-based *VSG* switching operates via RAD51-dependent and independent mechanisms.
